# NIA Division of Behavioral and Social Research

**DOI:** 10.1093/geroni/igab046.1047

**Published:** 2021-12-17

**Authors:** Lisbeth Nielsen

**Affiliations:** National Institute on Aging, Bethesda, Maryland, United States

## Abstract

Dr. Nielsen will discuss research priorities for the Division of Behavioral and Social Research. Dr. Nielsen will also be available for small group discussion.

